# AAV9‐mediated CIRP gene transfer protects against cardiac dysfunction and remodelling in a rat model of myocardial infarction

**DOI:** 10.1111/jcmm.70084

**Published:** 2024-10-14

**Authors:** Peng Zhong, Shuang Yang, Can Fang, Yanjun Li, Siwei Song, Minxiao Chen, Jingru Chen

**Affiliations:** ^1^ Department of Cardiology Renmin Hospital of Wuhan University Wuhan China; ^2^ Cardiovascular Research Institute of Wuhan University Wuhan China; ^3^ Hubei Key Laboratory of Cardiology Wuhan China; ^4^ Department of Pharmacology Renmin Hospital of Wuhan University Wuhan China

**Keywords:** cold‐inducible RNA‐binding protein (CIRP), heart failure, inflammation, myocardial infarction (MI)

## Abstract

Cold‐inducible RNA‐binding protein (CIRP) is a stress–response protein that has been shown to protect cardiomyocytes under a variety of stress conditions from apoptosis. Our recent study showed that the expression of CIRP protein in the heart was downregulated in patients with heart failure and an animal model of ischaemia heart failure, but its role in heart failure is still unknown. The present study aimed at evaluating the potential role of CIRP on the heart in an animal model of myocardial infarction (MI). MI model of rats was induced by the ligation of the left coronary artery. CIRP overexpression was mediated by direct intracardiac injection of adeno‐associated virus serotype 9 (AAV9) vectors carrying a CIRP coding sequence with a cardiac‐specific promoter before the induction of the MI model. The effects of CIRP elevation on MI‐induced heart were analysed through echocardiographic, pathological and molecular analysis. Our results showed that the intracardiac injection of AAV9 successfully mediated CIRP upregulation in cardiomyocytes. Upregulation of cardiac CIRP prevented MI‐induced cardiac dysfunction and adverse remodelling, coupled with the reduced inflammatory response in the heart. Collectively, these results demonstrated the beneficial role of intracellular CIRP on the heart and suggest that CIRP may be a therapeutic target in ischaemic heart disease.

## 1 INTRODUCTION

Acute myocardial infarction (MI) and heart failure (HF) that often follows are major causes of death and disability worldwide. Early reperfusion of myocardium by performing primary percutaneous coronary intervention is a highly efficient treatment to reduce the size of an acute MI. However, paradoxically, reperfusing the myocardium can subsequently result in cardiomyocyte death, referred to as myocardial reperfusion injury (MRI). MRI has been shown to contribute to 50% of final MI size.^1^ Although treatment has improved substantially, AMI still has 7% mortality rate within 1 year.^1^ Consequently, new therapeutic approaches are needed to limit MI size, prevent unfavourable myocardial remodelling and ultimately prevent the progress of post‐infarction HF.

CIRP (cold‐inducible RNA‐binding protein) is a stress‐responsive protein whose expression can be affected by various stressors including heat, oxidative stress, hypoxia and ultraviolet radiation. In response to stress, intracellular CIRP can migrate from the nucleus to the cytoplasm where it regulates mRNA stability by binding to the 3' UTR of its target mRNAs. Recent studies have shown that CIRP plays protective role in cardiomyocytes against apoptosis induced by stresses such as prolonged cardiac maintenance in vitro, chronic hypoxia and oxidative stimulation.^2^, ^3^, ^4^, ^5^ Interestingly, our recent studies showed that CIRP expression was downregulated in hearts from HF patients and animal models induced by myocardial infarction.^3^, ^6^ However, the effects of CIRP on HF associated with myocardial infarction remain poorly understood.

In this study, we examined the potential effects of CIRP overexpression in a rat model of MI‐induced HF. Our results confirmed that upregulation of cardiac CIRP prevented MI‐induced cardiac dysfunction and adverse structure remodelling, as well as reduced the inflammation in the heart. Taken together, our study supported a cardioprotective role of CIRP against myocardial infarction‐induced HF and suggest that CIRP is a promising therapeutic target for ischaemic cardiomyopathy.

## 2 METHODS

### 2.1 Preparation of AAV9

The pAAV constructs [pAAV‐cTnT‐CIRP‐P2A‐EGFP (pAAV‐CIRP) and pAAV‐cTnT‐MCS‐P2A‐EGFP (pAAV‐EGFP, MCS: multiple clone sites)], which encode the target genes under the control of chicken cardiac troponin T (cTNT) promoter, were produced and synthesized into the AAV9 capsids by the Hanbio Biotechnology (Shanghai, China) with a concentration at 6.5 × 10^12^ vg/mL.

### 2.2 In vivo AAV9 delivery

All animal handling protocols were approved by Renmin Hospital of Wuhan University Institutional Animal Care and Use Committee. Sprague–Dawley rats (6 weeks, male) were anesthetized with isoflurane, intubated and ventilated with a rodent ventilator. Then hearts were exposed when the chest was opened. The 200 μL AAV9‐EGFP or AAV‐CIRP at a concentration of 6.5 × 10^12^ vg/mL was injected directly and slowly into the left ventricle chamber, followed by the closure of the chest.

### 2.3 Establishment of MI model

Two weeks later, AAV9‐injected rats underwent another MI procedure via ligating left anterior descending coronary artery (LAD). Briefly, after anesthetized with sodium pentobarbital (0.05 g/kg, i.p.), rats were intubated and ventilated with an animal ventilator (ALC‐V8S). The lateral thoracotomy was performed between the fourth and fifth intercostal space. Subsequently, the LAD was ligated 2 mm below the root of left atrial appendage with 7.0 silk suture. MI criteria included pale stained myocardium and ST segment elevation on limb lead ECG. The sham group was subjected to sham surgery without MI. The incision in the rat's chest wall was sutured and the rat was placed on a heating pad and observed every 15 min until it fully recovered from anaesthesia.

### 2.4 Echocardiography study

At the end of the experiment, echocardiography was performed blindly by a technician to evaluate cardiac structure and function in each group. Rats were anesthetized with pentobarbital sodium (0.05 g/kg) intraperitoneally. M‐mode tracing was performed to determine left ventricular (LV) ejection fraction (LVEF), LV fractional shortening (FS), LV end‐diastolic inner diameter (LVIDd), LV end‐systolic inner diameter (LVIDs), LV end‐diastolic volume (LVEDV), LV end‐systolic volume (LVESV), LV posterior wall thickness in diastole (LVPWd), interventricular septum end‐diastolic thickness (IVSd) and interventricular septum end‐systolic thickness (IVSs). Parameters were measured by three times and averaged to minimize observer bias. At the end of the experiment, the rats were sacrificed under anaesthesia.

### 2.5 Histological analysis

The hearts were isolated and preserved in 4% paraformaldehyde for 48 h at 4°C for further embedding in paraffin LV tissues were cut into sections and then stained with fluorescein isothiocyanate‐labelled wheat germ agglutinin (WGA) or Sirius red to evaluate collagen deposition. Slides were visualized under a bright‐field microscope or a fluorescence microscope. Cell surface areas were measured and analysed using Image J software.

### 2.6 Immunofluorescent determination of EGFP expression in the heart

Evaluation of EGFP protein in the heart was performed by two ways. Fresh cryo‐preserved hearts were embedded in. O.C.T. compound. About 5‐μm cryosections were cut with a cryostat and were directly viewed under a fluorescence macroscope to visualize the EGFP fluorescence. In addition, tissue sections from the paraformaldehyde‐fixed hearts were incubated with primary antibodies for EGFP (ab184601, Abcam), and α‐actin (ab9465), followed by FITC‐conjugated or PE‐conjugated secondary antibodies incubation. Then the slides were visualized under a fluorescent macroscope.

### 2.7 Immunofluorescent determination of CIRP localization in the heart

Tissue sections from the paraformaldehyde‐fixed hearts with myocardial infarction were incubated with primary antibodies for CIRP (ab106230, Abcam), and α‐actin (ab9465), followed by FITC‐conjugated or PE‐conjugated secondary antibodies incubation. Then the slides were visualized under a fluorescent macroscope.

### 2.8 Western blots analysis

Total proteins were extracted from frozen LV samples in RIPA buffer upon the addition of protease inhibitors, and the protein concentrations was detected using BCA protein assay kit. Western blotting was performed to determine the expression of CIRP (ab106230, Abcam), collagen III (ab7778, Abcam), TNF‐a (ab9739, Abcam), IL‐1β (ab200478, Abcam), BNP (sc‐271185, Santa Cruz), cleaved‐caspase 3 (#9661, Cell Signaling) and Caspase 3 (#9662, Cell Signaling). The bands were visualized using ECL (Bio‐Rad) reagent. The protein contents were normalized to that of GAPDH (sc‐47724, Santa Cruz) as the endogenous control.

### 2.9 Real‐time quantitative PCR

Total RNA was isolated from various organs using TRIzol reagent (Invitrogen) and reverse‐transcribed to cDNA. Gene expression was analysed by quantitative PCR using Lightcycler 480 SYBR Green 1 Master Mix (Roche) and the Applied Biosystems VII7 (Life Technologies, USA) for tumour necrosis factor‐α (TNF‐α) and IL‐1β. The primer sequences have been shown in Table 1. All levels of reaction were normalized to that of GAPDH. As described previously,^7^ data were analysed using the 2^−∆∆Ct^ method.

**TABLE 1:** PCR primer sequence.

Gene	Species	Forward primer	Reverse primer
TNF‐α	Rat	CAGCCAGGAGGGAGAAC	GTATGAGAGGGACGGAACC
IL‐1β	Rat	CCCTTGACTTGGGCTGT	CGAGATGCTGCTGTGAGA
GAPDH	Rat	ATGGCTACAGCAACAGGGT	TTATGGGGTCTGGGATGG

### 2.10 Statistical analysis

In this study, statistical analyses were performed using GraphPad 5.0 software. All continuous data are expressed as mean ± SD and analysed by one‐way anova followed by Tukey's multiple comparison test. Statistical significance was set at a two‐tailed *p*‐value of <0.05.

## 3 RESULTS

### 3.1 Intracardiac injection of AAV9 successfully mediated cardiac CIRP upregulation

**FIGURE 1:**
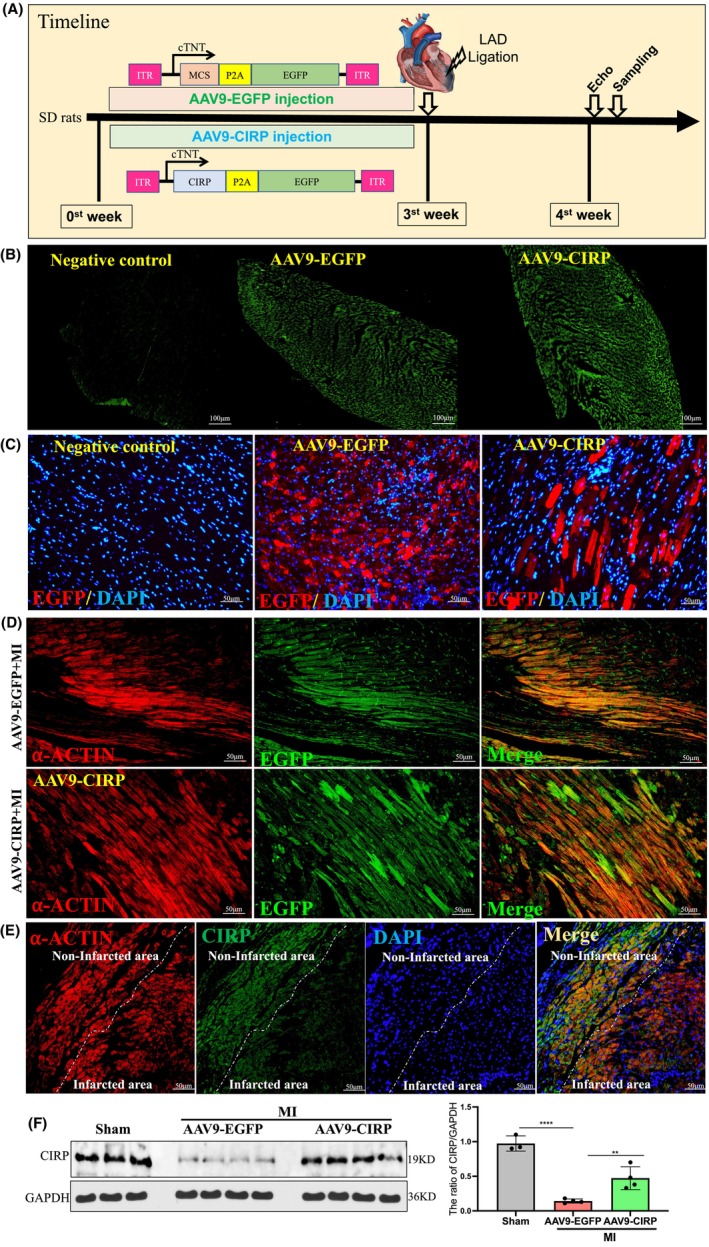
Evaluation of AAV9‐mediated target gene expression delivered by intracardial injection in rats. (A) The scheme of experimental procedure of the study. SD rats were intracardially injected with the AAV9 recombinant genome. Three weeks later, these AAV9‐injected rats were subjected to LAD ligation to establish an MI model for 1 week, followed by echocardiographic analysis and sample collection for histological and molecular analysis as described in the methods section. (B) Fluorescent microscopy confirmed robust expression of EGFP in the heart of AAV9‐injected rats. Constructs encoding cTNT‐MCS‐P2A‐EGFP and cTNT‐CIRP‐P2A‐EGFP are shown in the corresponding fluorescent images. (C) Immunofluorescence staining of EGFP in red colour to confirm the expression of EGFP in the AAV9‐injected heart. (D) Co‐staining of the heart section using both α‐action antibody (red fluorescence) and EGFP antibody (green fluorescence) in the MI+AAV9‐EGFP group and the MI+AAV9‐CIRP group. (E) Co‐staining of the heart section using both α‐action antibody (red fluorescence) and CIRP antibody (green fluorescence) in the infarcted heart. (F) Western blot analysis of CIRP protein level in the heart. *n* = 3–4 in each group, *p < 0.05; **p < 0.01; ****, *p* < 0.001.

AAVs are commonly used in biomedical and gene therapy research as gene transfer vectors. Cardiomyocytes can be efficiently transduced by AAV serotype 9 (AAV9). In this study, AAV9 vectors were used to express target genes under the control of the chicken cTNT promoter, which drives cardiomyocyte‐specific gene expression. The experimental procedure and AAV9 vector design used are shown in Figure 1A. To determine AAV9 gene delivery efficiency via intracardiac injection, the exogenous EGFP expression in cardiac tissue was examined at the end of experiment. As shown in Figure 1B, bright green fluorescence of EGFP was observed in rats treated with either AAV9‐CIRP or AAV9‐EGFP, indicating successful myocardial delivery of target genes. Furthermore, immunofluorescence staining confirmed EGFP presence in the heart (Figure 1C). Moreover, co‐staining using α‐action and EGFP antibodies also confirmed the presence of EGFP in cardiomyocytes. (Figure 1D). Co‐immunofluorescence staining of CIRP and α‐actin showed CIRP was primarily intracellular in both infarcted and non‐infarcted areas, with reduced intensity in infarcted areas (Figure 1E). We also found that the protein level of cardiac CIRP was downregulated in the MI+AAV9‐EGFP group when compared with sham group, which was significantly elevated in the MI+AAV9‐CIRP group (Figure 1F). Together, these results supported robust, cardiomyocyte‐specific CIRP expression via single intracardiac AAV9 administration.

### 3.2 AAV9‐mediated CIRP upregulation prevented cardiac dysfunction in MI rats

**FIGURE 2:**
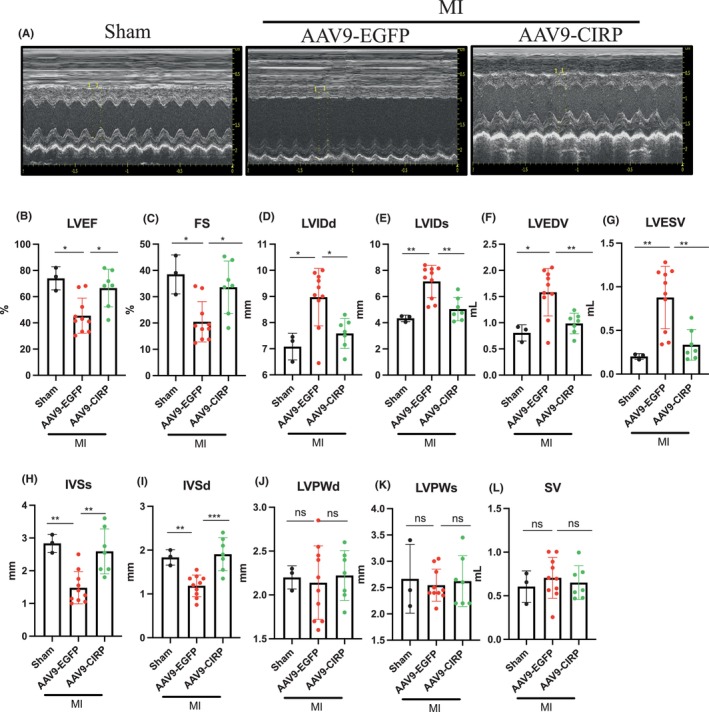
Effects of AAV9‐mediated CIRP elevation on cardiac function in MI rats. (A) Representative M‐mode echocardiograms at the end of the experiment. (B) LVEF: left ventricle ejection fraction. (C) FS: left ventricular fractional shortening. (D) LVIDd: left ventricular end‐diastolic inner diameter. (E) LVIDs: left ventricular end‐systolic inner diameter. (F) LVEDV: left ventricular end‐diastolic volume. (G) LVESV: left ventricular end‐systolic volume. (H) IVSs: interventricular septum end‐systolic thickness. (I) IVSd: interventricular septum end‐diastolic thickness. (J) LVPWd: left ventricular posterior wall end‐diastolic thickness. (K) LVPWs: left ventricular posterior wall end‐systolic thickness. (L) SV: stroke volume. *n* = 3–4 in each group, *p < 0.05, **p < 0.01; ***p < 0.001.

Seven days after MI, cardiac function was assessed in rats by echocardiography. As shown in Figure 2, LVEF and LVFS were markedly decreased in the MI+AAV9‐EGFP group when compared to those of the sham group. However, AAV9‐CIRP treatment significantly increased the LVEF and LVFS in the MI+AAV9‐CIRP group when compared with the MI+AAV9‐EGFP group. Furthermore, the MI+AAV9‐EGFP group had LV dilation, as evidenced by increases in LVEDV, LVESV, LVIDd and LVIDs, compared to the sham group. However, all these parameters were significantly improved in the MI+AAV9‐CIRP group. Interestingly, the IVSd and IVSs were also thinner in the MI+AAV9‐EGFP group than those in the sham group. However, IVSd and IVSs were significantly augmented in the MI+AAV9‐CIRP group. There were no differences in LVPWd and LVPWs among the three groups. In addition, no statistically significant differences were found between the sham group and MI+AAV9‐CIRP group in all echocardiographic parameters. These results suggested that upregulation of CIRP prevented MI‐induced cardiac dysfunction.

### 3.3 AAV9‐mediated CIRP upregulation prevented adverse cardiac remodelling in MI rats

**FIGURE 3:**
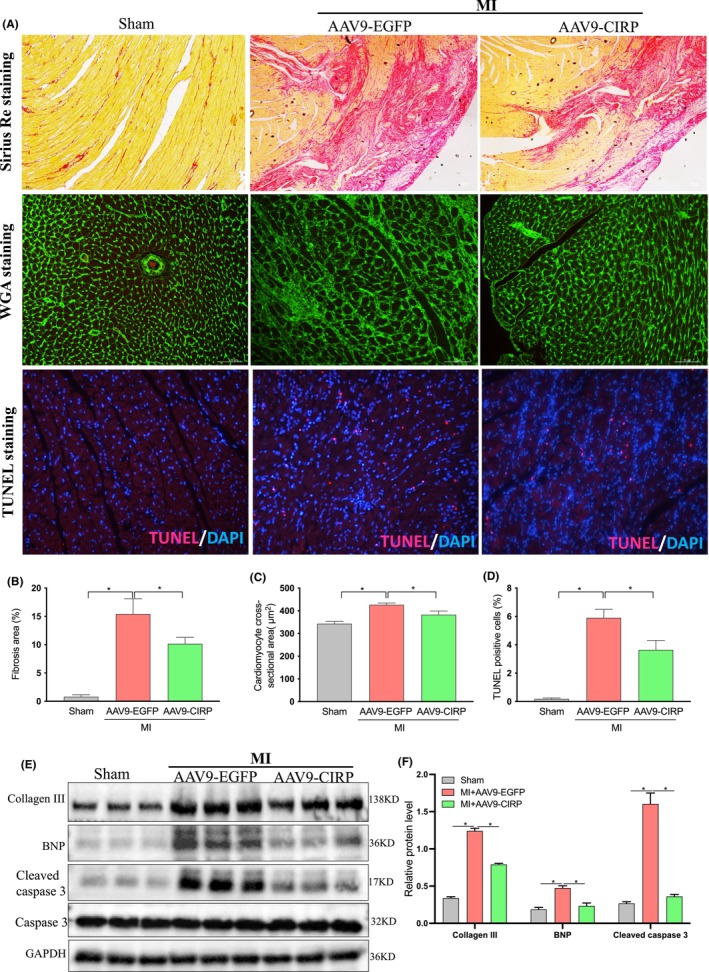
Effects of AAV9‐mediated CIRP elevation on adverse cardiac remodelling in MI rats. (A–D) Representative images of cardiac sections stained with Sirius red, WGA or TUNEL for the evaluation of cardiac fibrosis, cardiomyocyte size and cell apoptosis. (E and F) Western blot analysis of collagen III, BNP and cleaved‐caspase and caspase 3. GAPDH was used as an internal control. *n* = 3 in each group, *p < 0.05.

To assess the effects of CIRP overexpression on MI‐induced cardiac remodelling, we detected interstitial fibrosis, cardiac hypertrophy and cardiomyocyte apoptosis in hearts at 7 days after MI. As shown in Figure 3A,B, histological analysis of Sirius red‐stained cardiac sections revealed that the MI+AAV9‐EGFP group had more pronounced LV fibrosis accumulation in the marginal region of the infarcted heart compared with the MI+AAV9‐CIRP group. The results of the WGA immunofluorescence staining also showed a significantly increased cardiomyocyte size in the MI+AAV9‐EGFP group compared to that in the sham group, but a significant reduction of cardiomyocyte size in the MI+AAV9‐CIRP group (Figure 3A,C). TUNEL staining also revealed that a relatively lower level of TUNEL‐positive staining was detected in the MI+AAV9‐CIRP group compared to the MI+AAV9‐EGFP group (Figure 3A,D). Consistently, western analysis of the heart from the peri‐infarction area also showed more expression of markers of fibrosis, hypertrophic and apoptosis in the MI+AAV9‐EGFP group than that in the MI+AAV9‐CIRP group (Figure 3E,F). Collectively, these results suggest a beneficial effect of CIRP upregulation on MI‐induced adverse cardiac remodelling.

### 3.4 AAV9‐mediated CIRP upregulation reduced cardiac inflammation in MI rats

**FIGURE 4:**
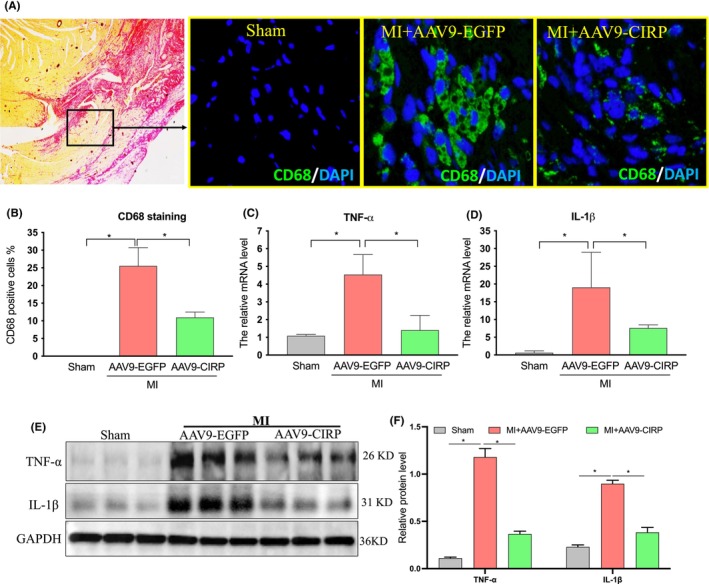
Effects of AAV9‐mediated CIRP elevation on cardiac inflammatory response in MI rats. (A) Representative images of heart tissue stained with CD68 fluorescent antibody (green) and DAPI (blue). (B) Quantitative analysis of CD68 staining in the peri‐infarction areas of the hearts. (C and D) The mRNA levels of TNF‐α and IL‐1β in the heart. GAPDH was used as a loading control. (E and F) The protein levels of TNF‐α and IL‐1β in the heart. *n* = 3 in each group, *p < 0.05.

It is well known that inflammation is essential for *healing* of the infarcted myocardium. However, excessive inflammation is harmful due to adverse pathological remodelling. To determine the potential impact of CIRP upregulation on the inflammatory response, we examined the infiltration of macrophages and the alterations of several inflammatory cytokines in the post‐infarcted hearts. As shown in Figure 4A,B, CD68^+^ macrophages were significantly increased at the peri‐infarct regions of the myocardium from the MI+AAV9‐EGFP group, whereas the number of the myocardial macrophages was lower in the MI+AAV9‐CIRP group than that in the MI+AAV9‐EGFP group. In addition, the mRNA levels of TNF‐α and IL‐1β were also elevated in the MI+AAV9‐EGFP hearts when compared to sham hearts, which were significantly attenuated by CIRP upregulation in MI+AAV9‐CIRP hearts (Figure 4C,D). Similar results were also found in the changes in the protein levels of these inflammatory genes (Figure 4E,F). Collectively, these results suggested an anti‐inflammatory effect of CIRP upregulation on the MI condition.

## 4 DISCUSSION

In this study, we reported, for the first time, that upregulation of cardiac CIRP by a single intracardial injection of AAV9 before ischaemic injury prevented MI‐induced cardiac dysfunction and improved adverse cardiac remodelling. Mechanistically, we observed that MI‐induced cardiac inflammatory response was greatly inhibited by CIRP upregulation. These results demonstrated a protective effect of CIRP on the heart and suggest that CIRP might be a promising therapeutic target in ischaemic heart diseases.

Recent studies have suggested that CIRP is a cardioprotective factor. For example, during prolonged hypothermic heart preservation in vitro, CIRP knockout rat hearts exhibited higher apoptosis and worse cardiac function versus CIRP transgenic hearts.^5^ Mechanistic studies revealed CIRP post‐transcriptionally increases CoQ_10_ biosynthesis proteins like COQ6 and COQ9, promoting ATP production and antioxidative capacity.^4^, ^5^ Additionally, CIRP knockout hearts showed reduced ubiquinone pathway proteins (such as COQ6 and COQ9), decreased concentration of CoQ_10_ and ATP production and aggravated oxidative stress during hypothermic cardiopulmonary bypass, which was prevented by CIRP overexpression or CoQ_10_ supplementation.^4^ Our previous study also demonstrated a protective role of CIRP against oxidative stress in vitro, as silencing of CIRP in cardiomyocytes promotes cell apoptosis in cardiac cells in response to H_2_O_2_ stimulation.^3^ Our further study identified Nrf2 as the downstream target of CIRP as silencing CIRP could reduce the expression of Nrf2 and it regulated antioxidant genes, leading to the compromise of cellular antioxidant capacity.^6^ In the present study, we further provide evidence that upregulation of cardiac CIRP levels could prevent the development of MI‐induced heart failure. Although the cardiac function recovery was very striking as showed in the LVEF and FS by the CIRP upregulation, the structural preservation of the heart as evidenced by the thickness of IVSs and IVSd was more impressive in the CIRP‐elevated heart. As the interventricular septum was mainly nourished by the left anterior descending coronary artery (LAD), the ligation of LAD would lead to significant infarction and thinning of the IVS. However, the IVS thickness either in systole or in diastole was remarkably preserved by CIRP elevation in response to MI, suggesting that elevation of CIRP could prevent cardiac infarction and limit infarction size. Collectively, these results demonstrated a cardioprotective role of intracellular CIRP in the heart.

While intracellular CIRP is beneficial in the heart, extracellular CIRP has been shown to be pro‐inflammatory and detrimental under inflammatory conditions. This conflicting intracellular versus extracellular role makes investigating CIRP complex. In this study, CIRP‐overexpressed heart subjected to myocardial infarction should release CIRP extracellularly where it would play a deleterious role. However, intracellular CIRP would play a protective role. As the overall effect of CIRP overexpression was beneficial, we think the protection is mainly attributed to intracellular CIRP. Additionally, co‐immunofluorescence staining of CIRP and α‐actin showed CIRP was primarily intracellular in both infarcted and non‐infarcted areas, with reduced intensity in infarcted areas. Collectively, these results indicate the protective effects of CIRP overexpression stem from increased intracellular CIRP levels, and targeting intracellular CIRP may be a therapeutic strategy for ischaemic heart diseases. Currently, there are two primary approaches to directly target intracellular CIRP. The simplest is using the chemical CIRP agonist zr17‐2, which elevates intracellular CIRP levels in vitro and in vivo by directly binding and inhibiting CIRP protein degradation. The second approach is gene therapy, as shown in this study.

Interestingly, CIRP upregulation markedly suppressed inflammation in the peri‐infarction region. Given the detrimental effects of an excessive and persistent pro‐inflammatory response to MI, the anti‐inflammatory effect of CIRP upregulation may contribute to the limitation of infarction size and the improvement of adverse cardiac remodelling in response to MI. However, recently it has been discovered that extracellular CIRP (eCIRP) occurs in inflammatory conditions and may play a pro‐inflammatory role. A number of factors have been reported to induce the release of CIRP into the extracellular space, including hypoxia, sepsis and haemorrhagic shock.^8^ In vitro, eCIRP has been identified as a damage‐associated molecular pattern (DAMP) to activate innate toll‐like receptor 4 (TLR4)/myeloid differentiation protein 2 (MD2)‐related pro‐inflammatory signal and thereby to trigger inflammation in macrophages.^9^, ^10^ In addition, eCIRP also functions as a new biologically active endogenous ligand of triggering receptor expressed on myeloid cells‐1 (TREM‐1) that incites inflammation in sepsis.^10^ These results suggest that eCIRP released either actively or passively during MI may contribute to the inflammatory response of the heart. However, herein, the inflammatory response had been inhibited in the MI+AVV9‐CIRP hearts when compared with that in the MI+AVV9‐EGFP heart, which possibly attributes to the reduced infarction size and injury by the intracellular CIRP elevation in cardiomyocytes, resulting in less release of pro‐inflammatory factors and recruitment of inflammatory cells in the infarcted areas. Therefore, the decreased inflammatory response in CIRP‐elevated heart may be a secondary response to the remarkable preservation of cardiac structure during MI condition.

Ample preclinical and clinical studies showed that AAVs have low immunogenicity, no pathogenicity and robust gene expression. Thus, AAVs has been positioned as the best vectors of choice for cardiac gene transfer. Herein, we showed that a single intracardial injection of AAV9 virus coupled with the cTNT promoter provides a specific and robust CIRP expression in the cardiomyocytes. The striking improvement of the cardiac structure and function in response to MI by CIRP gene therapy suggests that targeting CIRP by gene therapy may be a practical therapeutic strategy in ischaemic heart diseases.

## 5 LIMITATIONS

In the present study, the baseline effect of CIRP overexpression is not assessed. As previous published works have showed that at baseline CIRP transgenic rats showed no abnormalities in cardiac function or pathological changes as evidenced by echocardiographic and histological analysis,^4^ we think that the AAV9‐mediated CIRP overexpression may also have no impact on the heart, which needs further verification.

In summary, this study demonstrated that the elevation of CIRP in cardiomyocytes by intracardiac delivery of the AAV9 carrying CIRP gene is effective in preventing MI‐induced cardiac dysfunction and adverse remodelling. These results support a cardioprotective effect of CIRP in the heart and CIRP may be a therapeutic target in ischaemic disease.

## AUTHOR CONTRIBUTIONS

**Peng Zhong:** Conceptualization (equal); writing – original draft (equal). **Shuang Yang:** Investigation (equal). **Can Fang:** Investigation (equal). **Yanjun Li:** Investigation (equal). **Siwei Song:** Investigation (equal). **Minxiao Chen:** Investigation (equal). **Jingru Chen:** Conceptualization (equal).

## CONFLICT OF INTEREST STATEMENT

The authors declare no competing financial interests or conflicts concerning the work described.

## Data Availability

The data that support the findings of this study are available from the corresponding author upon reasonable request.
